# Moderate Weight Reduction in an Outpatient Obesity Intervention Program Significantly Reduces Insulin Resistance and Risk Factors for Cardiovascular Disease in Severely Obese Adolescents

**DOI:** 10.1155/2011/541021

**Published:** 2011-08-22

**Authors:** J. Grulich-Henn, S. Lichtenstein, F. Hörster, G. F. Hoffmann, P. P. Nawroth, A. Hamann

**Affiliations:** ^1^Department of Pediatrics, University of Heidelberg, INF 430, 69120 Heidelberg, Germany; ^2^Division of Endocrinology and Metabolism, Department of Internal Medicine, INF 410, 69120 Heidelberg, Germany; ^3^Diabetes-Clinic Bad Nauheim, Kerckhoff Campus for Cardiovascular Medicine, Ludwigstrasse 37-39, 61231 Bad Nauheim, Germany

## Abstract

*Background*. Metabolic risk factors like insulin resistance and dyslipidemia are frequently observed in severly obese children. We investigated the hypothesis that moderate weight reduction by a low-threshold intervention is already able to reduce insulin resistance and cardiovascular risk factors in severely obese children. *Methods*. A group of 58 severely obese children and adolescents between 8 and 17 years participating in a six-month-long outpatient program was studied before and after treatment. The program included behavioral treatment, dietary education and specific physical training. Metabolic parameters were measured in the fasting state, insulin resistance was evaluated in an oral glucose tolerance test. *Results*. Mean standard deviation score of the body mass index (SDS-BMI) in the study group dropped significantly from +2.5 ± 0.5 to 2.3 ± 0.6 (*P* < 0.0001) after participation in the program. A significant decrease was observed in HOMA (6.3 ± 4.2 versus 4.9 ± 2.4, *P* < 0.03, and in peak insulin levels (232.7 ± 132.4 versus 179.2 ± 73.3 *μ*U/mL, *P* < 0.006). Significant reductions were also observed in mean levels of hemoglobin A_1c_, total cholesterol and LDL cholesterol. *Conclusions*. These data demonstrate that already moderate weight reduction is able to decrease insulin resistance and dyslipidemia in severely obese children and adolescents.

## 1. Introduction

During the last decade a steady rise in the prevalence of obesity in children and adolescents has been observed worldwide [1, 2] and in several age-groups the extent of obesity was also on the increase. Concurrently certain diseases, characteristically occurring in middle-age adults in association with enhanced body fat mass (e.g., metabolic syndrome and type 2 diabetes), have emerged in children and adolescents with severe obesity, suggesting a coherence between both trends [3, 4]. Likewise, further investigations have shown that obese children and adolescents have a high risk between 30 and 80% for the persistence of overweight into adulthood, and that morbidity and mortality are higher in those obese adults who became overweight during childhood compared to those whose weight-gain evolved later in life [5]. On the other hand epidemiological studies showed that the majority of severely obese adults became overweight when they were still children. A tremendous increase in obesity-related morbidity and furthermore an immense rise in the medical costs associated with it, are to be expected, if this trend continues [6]. For instance, at present type 2 diabetes mellitus is already predominant in some pediatric diabetes centers in the USA due to severe obesity [7]. In Germany more and more obese adolescents are also affected by type 2 diabetes mellitus [8].

Insulin resistance is a common feature of obesity in the young and precedes impaired glucose tolerance and type 2 diabetes mellitus. In studies performed at our center, insulin resistance was observed in approximately 70% of severely obese children and adolescents. These data are in accordance with similar studies performed in other European countries and in the United States [9, 10]. It is imperative that we address the obesity epidemic in young people by the development of specific programs; failure to do so could have severe consequences for the economy. The parents of obese children are usually also markedly obese. This feature may to a certain degree be due to the transmission of adverse parental genes, but more often it can be attributed to the lifestyle transferred from parents on their children [11]. It is evident that the most promising way to avoid further morbidity is the treatment of the problem in the whole family [12]. Meanwhile a variety of studies have demonstrated that multimodal lifestyle interventions including behavioral measures, exercise programs, and dietary advicing are effective in the treatment of obese children [1, 13]. Not surprisingly, more intensive programs were more effective in reducing overweight in children. However, many obese children are not able to participate in such intensive programs. Low-threshold obesity intervention programs facilitate participation for families, since the children can continue their daily life routines. Reinehr and Andler have shown in their study that a reduction of SDS-BMI of at least 0.5 is required for an improvement of atherogenic profile and insulin resistance [14]. Therefore the aim of the present study was to investigate whether a low-threshold obesity program which achieves moderate weight reduction is able to alter cardiovascular risk factors in severe obese adolescents.

## 2. Materials and Methods

### 2.1. Patients

The study population consisted of 32 girls and 26 boys between the ages of 8 and 17 with a mean age of 12.6 ± 2.2 years with profound obesity (body mass index >97th percentile for ages according to standards for German children [15]). Body weight was measured in the morning in a fasting state using a digital scale to the nearest 0.1 kg. Height was measured using a wall-mounted stadiometer. Body mass index (BMI) was calculated (weight in kilograms divided by the body height in square meters), and the standard deviation score of the BMI (SDS-BMI) was calculated according to Cole [16]. Informed consent of the patients and their parents was given before the study began.

### 2.2. Oral Glucose-Tolerance Test

Participants were studied at 8.00 to 8.30 a.m. after a 12-hour overnight fasting period. An antecubital intravenous catheter was inserted for blood sampling and maintained open by infusion of physiological saline solution. All participants rested during the test. Baseline blood-samples were obtained, and thereafter the participants received orally a standardized glucose solution (Dextro-OGT, Roche, Basel, Switzerland) in a dose of 1.75 g per kilogram of body weight up to a maximum of 75 g. Blood samples were collected at 30 to 120 minute intervals.

### 2.3. Biochemical Assays

Blood samples for measurements of glucose were collected in sodium fluoride containing tubes, and glucose was measured by the glucose-oxidase method. Insulin and leptin were measured by radio-immunoassay. Total cholesterol, HDL cholesterol, LDL cholesterol, and hemoglobin A_1c_ (HbA_1c_) were measured from fasting blood samplings. All biochemical measurements were performed in an accredited hospital laboratory.

### 2.4. Intervention Program

Medical examination and psychological evaluation were performed before entry into the program. Children with severe medical disorders besides obesity or with severe psychosocial impairments were excluded. Our six-month program consists of

- nutritional consultation with the family members (6 monthly sessions),
- cognitive-behavioral training in groups of 8–10 participants (6 monthly sessions),
- physical activity programs in groups of 8–10 participants (24 weekly lessons).

All procedures were carried out by a nutritionist, a psychologist, and physiotherapists in our institution. During the program, the participants maintained their normal daily routine, and visited school as usual. Children and adolescents participated once a week in a specific physical training program for obese adolescents. The exercise lessons consisted of one-hour “indoor cycling” in groups of 8 to 10 children on specific stationary bikes under instruction. In addition, children and their families were encouraged to increase their physical activities in daily life, for example, by watching fewer TV programs or driving less or by doing more active leisure time activities. In the nutrition counseling sessions the participants and their parents were guided by the nutritionist to change their eating habits to a low fat and healthy diet according to the national recommendations of the Research Institute of Child Nutrition Dortmund (http://www.fke-do.de) and the German Nutrition Society (http://www.dge.de/). Particularly the amount of mono- and disaccharides in sweets or sweetened drinks needed to be limited in many subjects. Other dietetic problems like generally too large amounts, poor diets without “five a day” or not enough diaries or drinking amounts are some other items of the nutrition sessions [17]. The change of family eating patterns was recommended and trained in terms of flexible control [18]. The cognitive-behavioral training was performed according to a program developed by Warschburger et al. [19].

### 2.5. Statistical Analysis and Calculations

The homeostasis model assessment (HOMA) was used as one measure of insulin resistance and was calculated as fasting glucose (mmol/L) × fasting insulin (*μ*U/mL)/22.5) [20]. Nonparametric procedures were used for all comparisons. Mann-Whitney test was used for comparison of sex- and age-specific differences. Wilcoxon signed rank test was used for comparison before and after treatment. The StatView-statistical analysis program (SAS-Europe, Heidelberg) was used for statistical analysis. A probability value of <0.05 was considered significant.

## 3. Results

**Figure 1:**
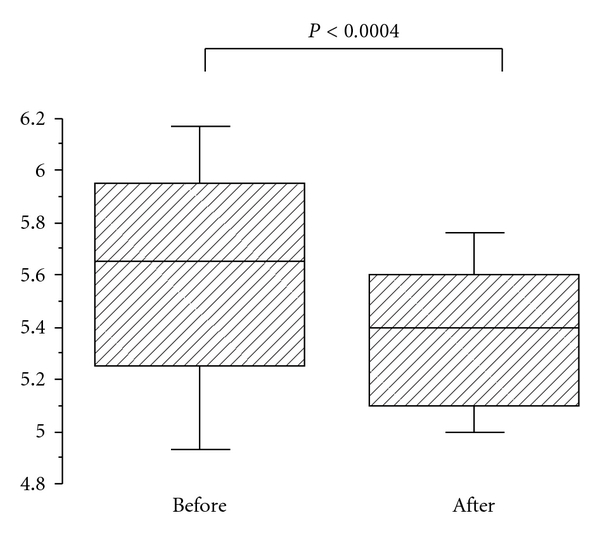
Hemoglobin A_1c_ before and after weight loss. Hemoglobin A_1c_  (%) was measured before and after weight loss as described in the methods. Box plots represent the 10th, 25th, 50th, 75th, and 90th percentiles. Statistical analysis was performed by Wilcoxon signed rank test.

**Figure 2:**
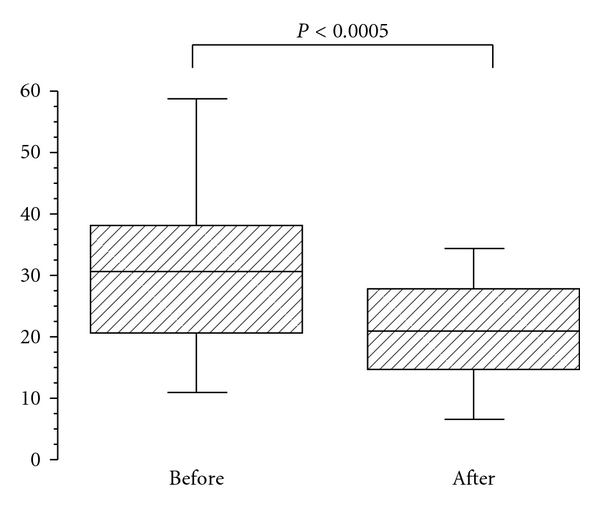
Leptin levels before and after weight loss. Leptin (ng/mL) was measured before and after weight loss as described in the methods. Box plots represent the 10th, 25th, 50th, 75th, and 90th percentiles. Statistical analysis was performed by Wilcoxon signed rank test.

Medical examination was performed before and at the end of the program. Mean BMI of the study group was 30.7 kg/m^2^ ± 5.0 before treatment and 29.5 kg/m^2^ ± 4.7 after treatment (Table 1). For statistical analysis SDS-BMI was used, since BMI normal values are age-dependent during childhood and adolescence. Mean SDS-BMI of the study group before treatment was +2.5 ± 0.5. There were no statistical significant differences in SDS-BMI, or in laboratory values between girls and boys (data not shown), and therefore boys and girls were not analyzed separately. SDS-BMI dropped significantly to 2.3 ± 0.6 after weight reduction (*P* < 0.0001) as shown in Table 1. Fasting glucose levels were within the normal range in all participants, and none of the participants had impaired glucose tolerance, defined by 2-hour glucose levels >7.7 mmol/L. However, there was a marked insulin resistance determined by fasting insulin levels, peak insulin levels, and homeostasis model assessment (HOMA). Mean HOMA was 6.3 ± 4.2 before treatment and dropped significantly to 4.9 ± 2.4 after treatment (Table 2). Mean fasting insulin was 28.3 ± 16.5 *μ*U/mL before treatment and decreased significantly to 23.8 ± 10.6 *μ*U/mL after treatment as shown in Table 2. Peak insulin levels were elevated and also decreased significantly after participation in the program as shown in Table 2. Furthermore, the area under the curve for insulin in the oGTT (AUCins) was also significantly reduced after weight reduction (data not shown). We noted a good correlation between HOMA and peak insulin levels (*r* = 0.81, *P* < 0.0001). Fasting glucose levels showed no significant differences before and after treatment (4.7 ± 0.6 versus 4.7 ± 0.5 mmol/L, whereas peak glucose levels in the oGTT were seen at 60 minutes and were significantly higher before weight loss (6.9 ± 1.4 mmol/L versus 6.3 ± mmol/L, *P* < 0.03; Table 2). The mean HbA_1c_-level was 5.6 ± 0.4% before treatment and decreased significantly to 5.4 ± 0.3 (*P* < 0.0004) as shown in Figure 1. A significant decrease was also noted in leptin levels (Figure 2). We noted a highly significant correlation between leptin levels and HOMA (*r* = 0.433, *P* > 0.0001) as well as between leptin levels and peak insulin levels (*r* = 0.61, *P* < 0.006) before treatment. HOMA levels above 4, considered to represent a high risk for the development of metabolic syndrome, were observed in 66% before and in 54% after weight reduction (Table 3). None of the participants had impaired glucose tolerance of diabetes mellitus type 2, according to WHO standards. However, we observed HbA_1c_ values above the normal range in 5 adolescents (8.6%) before treatment. At the end of the treatment period all five individuals had HbA_1c_ levels within the normal range (Table 3). 

Furthermore, lipid and lipoprotein fractions were analyzed before and after weight reduction. As shown in Table 2, mean levels of total cholesterol and LDL cholesterol decreased significantly after weight reduction, while no significant difference was noted in mean triglyceride levels. In contrast to these favorable changes, HDL cholesterol levels also decreased during the treatment period (1.1 ± 0.2 versus 0.9 ± 0.2 mmol/L, *P* < 0.002). Since mean levels might not reflect the risk profile, we also determined the percentage of participants with levels within pathological ranges before and after weight reduction. Hypercholesterolemia, defined as total cholesterol levels >5.0 mmol/L, was noted in 25% of participants before treatment, and in 18% after treatment. LDL levels >3.5 mmol/L were observed in 16% before and only in 10% after weight reduction. Elevated triglyceride levels (>1.6 mmol/L) were seen in about 30% before and after weight reduction (Table 3). Decreased HDL levels (<0.9 mmol/L) were seen in 27% before treatment and increased up to 51% after treatment.

## 4. Discussion

The present study demonstrates that a low threshold obesity intervention program is effective in reducing BMI in severely obese children and adolescents. Besides the positive impact on obesity, we could demonstrate that risk factors for cardiovascular diseases were also significanty lowered after participation in the intervention program. The program consisted of nutritional consulting, behavioral training, and physical activity programs. All three elements are considered to be important for the success of an obesity program [21]. Nutritional education was an important element of the program, and it became evident that repeated sessions would be necessary to change the eating habits of the families. This observation is in accordance with a long-term follow-up study of overweight children showing that single consultation sessions are not sufficient to achieve weight loss [22]. The nutritional consultation was performed on a family basis in order to give individual advice on the families' eating habits. It was important that the parent mainly responsible for preparing the meals attended these sessions. The behavioral training and physical activity programs were performed in groups of 8–10 participants without other family members. Although most participants were antipathy to the cognitive-behavioral training at the beginning, the majority gave positive feedback at the end of the program. The important role of physical activity in weight reduction programs has been demonstrated [23]. The physical activity program was well accepted by most of the participants.

Impaired glucose tolerance, insulin resistance, and a higher prevalence of risk factors for cardiovascular diseases are common metabolic features of childhood obesity [9, 10, 24]. However, in contrast to adults, it is to date not possible to correlate the degree of childhood obesity to the associated health consequences, because clear-cut evidence for threshold values is missing. Compared to more intensive in-patient programs the weight reduction achieved by our program was moderate. Nevertheless profound improvements in the metabolic situation of the children and adolescents were observed. Marked insulin resistance was observed in the study group before treatment. Several models for measuring insulin resistance have been proposed, but to date no general agreement has been made on the preferential model [25]. The thresholds for insulin resistance in childhood and adolescents have been adapted from those designed for adults. We chose HOMA, fasting insulin levels, and peak insulin levels as measures for insulin resistance, since these parameters have been used in comparable studies performed in children and adolescents [10, 14, 25]. Participation in the program led to a marked reduction of insulin resistance as shown in Table 2. Only participants who participated in the whole program were included into the study. Long et al. demonstrated in adults that moderate weight reduction of 5–10% can prevent progression of impaired glucose tolerance in severely obese subjects [26]. Reinehr and Andler published improvements in HOMA and in lipid profiles after participation in a more intensive weight reduction program in children [14]. HbA_1c_-levels, reflecting long-term blood sugar regulation, were within the normal range in most patients, and only slightly elevated in 5 individuals. A significant decrease in HbA_1c_ was observed after weight reduction, despite the fact that a carbohydrate-enriched diet was recommended during the program. The reduction in mean HbA_1c_ might be an indicator of improved carbohydrate metabolism and a consequence of the reduction of insulin resistance. Such an effect of weight reduction on HbA_1c_ has not been reported before. According to recent data, even an increasing HbA_1c_-level above 5% is already associated with elevated cardiovascular risk in adults. Therefore, the changes in HbA_1c_ we observed in our study could be quite meaningful in the long term [27]. 

Plasma leptin levels are positively correlated with BMI, and several studies demonstrated that obese subjects exhibit leptin resistance [28]. Associations between insulin resistance and leptin levels have been reported in children [29]. It was therefore not surprising that leptin levels decreased due to weight reduction in the present study. Arslanian et al. showed a significant correlation between leptin levels and fasting insulin levels [30]; an even more pronounced correlation could be demonstrated between HOMA or peak insulin levels on one hand and leptin levels on the other. It seems that leptin resistance and insulin resistance in obese children and adolescents are closely related and are possibly regulated in the same sense. 

Dyslipidemia is a frequent feature of obesity in adults as well as in children [31–33]. Mean levels of plasma lipids were within the normal range before treatment (see Table 2), but dyslipidemia defined by elevated total cholesterol, LDL cholesterol, triglycerides, and reduced HDL cholesterol was seen in almost 25% of the study group before treatment. A significant reduction was achieved in total cholesterol and LDL cholesterol by weight reduction, while triglycerides showed no significant changes. These findings are in accordance with data by Reinehr and Andler who also demonstrated significant changes in LDL cholesterol, and to a lesser degree in triglycerides, while total cholesterol was not measured in that study [14]. HDL cholesterol levels decreased also during the intervention. This phenomenon appears to be temporary due to the low calorie and low fat diet and has been described in adults before [34]. Reinehr and Andler also reported that HDL cholesterol decreased during the period of dieting but increased again after stabilization of the body weight [14]. A recent systematic review showed improved HDL-cholesterol profiles after weight loss in most studies [35]. The reduction in HDL-cholesterol in the present study might be due to the fact that low fat diets were recommended in our program. 

In summary we could demonstrate that already moderate weight reduction has beneficial consequences on multiple factors involved in the development of the metabolic syndrome and thereby may help to prevent the development of diabetes mellitus type 2 in these individuals.

## Figures and Tables

**Table 1:** Anthropometric data of the study group before and after participation in the obesity intervention program.

	Before treatment			After treatment			*P*
	Min	Mean	Max	Min	Mean	Max	
Height (cm)	125	163.1	187.8	131.4	164.8	188.5	<0.0001
Weight (kg)	35.3	84.5	143.5	37.4	83.3	146.3	n.s.
BMI	22.7	30.7	44.3	21.7	29.5	44.5	<0.0001
SDS-BMI	+1.9	+2.5	+3.8	+1.3	+2.3	+3.7	<0.0001

**Table 2:** Metabolic characteristics before and after participation in the obesity intervention program. Data are shown as means ± SD.

	Before treatment	After treatment	*P*
Fasting insulin (*μ*U/mL)	28.3 ± 16.5	23.8 ± 10.6	<0.03
Peak insulin (*μ*U/mL)	232.7 ± 132.4	179.2 ± 73.3	<0.006
HOMA	6.3 ± 4.2	4.9 ± 2.4	<0.03
Fasting glucose (mmol/L)	4.7 ± 0.6	4.7 ± 0.5	n.s.
Peak glucose (mmol/L)	6.9 ± 1.4	6.3 ± 1.4	<0.03
Total cholesterol (mmol/L)	4.5 ± 0.9	4.1 ± 0.9	<0.0001
HDL cholesterol (mmol/L)	1.1 ± 0.2	0.9 ± 0.2	<0.002
LDL cholesterol (mmol/L)	2.9 ± 0.8	2.5 ± 0.7	<0.004
Triglycerides (mmol/L)	1.3 ± 0.6	1.2 ± 0.5	n.s.

**Table 3:** Patients at risk before and after participation in the obesity intervention program.

	Before treatment	After treatment
Total cholesterol >5 mmol/L	25%	18%
LDL cholesterol >3.5 mmol/L	16%	10%
HDL cholesterol <0.9 mmol/L	27%	51%
Triglycerides >1.6 mmol/L	30%	30%
HOMA >4	66%	54%
HbA_1c_ >6.1%	10%	0%
